# Using Long-Term Volunteer Records to Examine Dormouse (* Muscardinus avellanarius *) Nestbox Selection

**DOI:** 10.1371/journal.pone.0067986

**Published:** 2013-06-27

**Authors:** Rachel L. Williams, Anne E. Goodenough, Adam G. Hart, Richard Stafford

**Affiliations:** 1 School of Natural and Social Sciences, University of Gloucestershire, Cheltenham, United Kingdom; 2 School of Applied Sciences, Bournemouth University, Fern Barrow, Poole, United Kingdom; University of Western Ontario, Canada

## Abstract

Within ecology, there are unanswered questions about species-habitat interactions, which could potentially be resolved by a pragmatic analysis of a long-term volunteer-collected dataset. Here, we analysed 18 years of volunteer-collected data from a UK dormouse nestbox monitoring programme to determine the influence of habitat variables on nestbox choice by common dormice ( *Muscardinus* *avellanarius* ). We measured a range of habitat variables in a coppiced woodland in Gloucestershire, UK, and analysed these in relation to dormouse nestbox occupancy records (by dormice, other small mammals, and birds) collected by volunteers. While some characteristics of the woodland had changed over 18 years, simple transformation of the data and interpretation of the results indicated that the dataset was informative. Using stepwise regressions, multiple environmental and ecological factors were found to determine nestbox selection. Distance from the edge of the wood was the most influential (this did not change over 18 years), with boxes in the woodland interior being selected preferentially. There was a significant negative relationship with the presence of ferns (indicative of damp shady conditions). The presence of oak (a long-lived species), and the clumped structural complexity of the canopy were also important factors in the final model. There was no evidence of competition between dormice and birds or other mammals. The results provide greater understanding of artificial dormouse nest-site requirements and indicate that, in terms of habitat selection, long-term volunteer-collected datasets contribute usefully to understanding the requirements of species with an important conservation status.

## Introduction

Many animals, both invertebrate and vertebrate, build nests (e.g. stingless bees *Trigona* *spinipes* [1]; grass-cutting ants *Atta* *vollenweideri* [2]; chimpanzees *Pan* *troglodytes* [3]; great tits *Parus* *major* [4]). Selecting a suitable nest-site is important as it provides shelter from predators or adverse weather conditions, and increases fitness and survival of young [5–7]. Most nest-building birds, for example, invest considerable time and energy choosing their nest-site because certain sites greatly influence reproductive success [8] and the same is true for large mammals (e.g. badgers *Meles* *meles* [9]), and for many small mammal species (e.g. [5,10,11]). Knowledge of nest-site requirements is essential for the conservation of rare or specialist species [7,12], especially where nest-site availability limits population sizes, as has been observed in a variety of arboreal mammals (e.g. grey mouse lemurs *Microcebus* *murinus* [13]; northern flying squirrels *Glaucomys* *sabrinus* [14]; greater gliders *Petauroides* *volans* [15]; common dormice *Muscardinus* *avellanarius* [16]).

In the UK, a lack of appropriate woodland management and habitat fragmentation has resulted in the reduction of suitable habitat for dormice, at the edge of their range, leading to extirpations [17]. As a result, and despite legal protection, dormouse distribution has reduced by more than half since the 19^th^ century, and the species is now of conservation concern in the UK [18]. Dormouse nesting ecology is difficult to study because dormice are cryptic, nocturnal and arboreal; their natural nests are difficult to locate as they are usually concealed in thick foliage or in tree cavities, and may be as high as 15 m in the canopy [17,19]. This makes studies relying on natural nest-sites logistically challenging, or even misleading, because of the high risk of not finding nests [20]. Nestbox occupation data provide an opportunity to estimate relative abundance and distribution of dormice with minimal labour [21]. Dormice are found in nestboxes from mid-May to October, and are known to use them across their range, thereby allowing the comparison of findings across similar studies [22]. Nestboxes also benefit dormouse conservation. Bright and Morris [20] conducted a radio-tracking survey and found that artificial nestboxes were by far the most frequently used nest-sites compared to natural nests. They argued that, where nestboxes are present, almost the whole population would use them, and providing nestboxes appeared to double the number of dormice present in an area [20]. Some cavity-nesting bird species such as blue and great tits are known to use artificial nestboxes almost exclusively when they are available, and numerous studies have benefited from the study of these species in nestboxes [23]. As dormice also readily breed in nestboxes [24], this also allows the study of their breeding ecology. Both male and female dormice use nestboxes, and they can be found either singly or in groups of two or more (e.g. male-female breeding pairs, groups of juveniles, mothers with litters) and this fluctuates depending on the time of year. Dormice can have several litters per year, although exact numbers of litters and young per litter differ across their range [22] (note that two litters per year were commonly found in some nestboxes at the present study site; one in early summer and one in the autumn). Any findings that relate habitat features to nestbox preference or breeding success in nestboxes could therefore easily be used in an applied sense (e.g. changing nestbox location) and may have more immediate conservation implications than findings relating to habitat features in natural nest-sites (because these cannot be moved), although factors influencing the selection of natural and artificial sites may not be identical.

There is a growing focus on long-term volunteer-collected datasets in ecology [25,26] because volunteer-run programmes provide large quantities of data at minimal cost [27,28]. Deploying a team of volunteers can also save substantial amounts of time compared to using professional ecologists [27]. In the UK, many conservation organisations rely heavily on volunteers to collect data (e.g. the British Trust for Ornithology BTO, the Royal Society for the Protection of Birds RSPB, the People’s Trust for Endangered Species PTES, the Mammal Society, the Marine Conservation Society, the Wildlife Trusts and the Bat Conservation Trust), however, volunteer-collected data are often questioned because they lack the rigour and precision of scientific studies (e.g. [29]).

The dormouse is a popular and charismatic species in the UK. Currently, over 1,000 volunteers participate in the National Dormouse Monitoring Programme (NDMP) run jointly by the PTES and Natural England. These volunteers have been submitting records since 1988, and in 2011, there were 305 sites involved in the scheme (with some annual variation – S. Sharafi, PTES, pers. comm.). Volunteers are required to check nestboxes at a site at least twice a year (May/June and Sept/Oct) to monitor evidence of dormouse occupation. The records are analysed by the PTES to estimate national trends in dormouse numbers and distribution.

Understanding breeding dormouse population nestbox requirements is crucial if nestboxes are to be maximally effective for conservation. Using long-term (18-year) volunteer-collected data collected as part of the NDMP, this study: (1) tests whether dormice actively choose (rather than randomly occupy) nestboxes; (2) examines some of the biotic and abiotic factors responsible for this selection; and (3) provides recommendations on using large volunteer datasets, discussing the attributes and limitations such datasets present.

## Methods

### Site Description

This study was undertaken at Midger Wood Nature Reserve (51° 36' 15.8", 2° 17' 26.9"), a 9 ha site in Gloucestershire, UK, managed by the Gloucestershire Wildlife Trust. The site is an ancient semi-natural coppiced woodland, dominated by ash ( *Fraxinus* *excelsior* ) with some Pedunculate oak ( *Quercus* *robur* ) and beech ( *Fagus* *sylvatica* ), with an understory of hazel ( *Corylus* *avellana* ), hawthorn ( *Crataegus* *monogyna* ), and holly ( *Illex* *aquifolium* ) [30].

### Data Collection

The presence of dormice, other small mammals (combining records for woodmice *Apodemus* *sylvaticus* , yellow-necked mice *Apodemus* *flavicollis* , and shrews *Sorex* spp.), and birds (mainly blue tits *Cyanistes* *caeruleus* and great tits *Parus* *major* ) was recorded monthly from April to November in 97 wooden dormouse nestboxes between 1994 and 2011 inclusive (no other species were found, and there was no indication of grey squirrels *Sciurus* *carolinensis* entering the nestboxes to compete with, or depredate, dormice). Nestboxes were located at chest height, and were distributed along transects across the hazel coppice coupes of the wood, such that they were at least 20 m apart, in accordance with NDMP guidelines [31] (note that the number and location of the nestboxes remained the same over the 18 year period). Although the nestboxes were situated substantially lower than the potential height of natural nest-sites for logistical reasons (following NDMP guidelines), there is no evidence to suggest that this makes them less attractive to dormice than higher natural nest-sites (see 20). Additionally, Sara et al. [32] found no significant difference between nestboxes placed at 1.5 m, 3 m and 5 m above ground. Nestbox monitoring was undertaken by volunteers for Gloucestershire Wildlife Trust (GWT), who manage the site. New volunteers were trained by long-term volunteers who accompanied them until they had enough experience to qualify for a dormouse handling license (a legal requirement in the UK [33]). Nestboxes measured 140x140 mm at the base, had a slanted roof with a mid-point height of 160 mm and a rear entrance hole of 30 mm in diameter, and were fixed to trees at chest height. Volunteer-collected data included presence or absence of nests and the number of individuals found in the nestbox during the survey. Volunteers did not search for natural nest-sites, since 1) this is not a requirement of the National Dormouse Monitoring Programme; and 2) there would have been considerable difficulty locating natural nests [17,19]. Summary data can be requested from the Gloucestershire Centre for Environmental Records (GCER). Dormouse occupation of nestboxes was relatively low, with an average of 7.3% of boxes occupied in any given year (S.D. = 3.3, minimum 2%, maximum 13%).

Volunteers removed nests and cleaned nestboxes at the end of winter each year unless the nestbox contained a dry, intact dormouse nest, as the volunteers hoped that this may encourage dormice to re-use the nestbox in the following year. Since dormouse nests were sometimes left over successive years, this variable could not be assured to be independent between years, and certainly not between monthly surveys. Furthermore, historic records showed that dormice were occasionally absent from nestboxes even when recently-made nests were found during a survey. As such, the presence of individuals in a nestbox at any point during the year was used as a dependent variable, since this removed the confounding results of nests being present between successive recordings, but also accounted for the lower likelihood of sightings of individuals compared to nests (this variable is termed dormouse occupancy).

The percentage of occupancy for each nestbox was calculated over the 18-year period (e.g. 9 years of occupancy = 50% occupancy). We hypothesised that leaving nests in nestboxes over successive years may have an influence on dormouse nestbox selection, alongside habitat variables surrounding the nestbox. To remove this effect, dormouse nestbox selection was also examined by treating dormouse nests as a binary variable (whereby nestboxes that had contained a nest at any time over the 18-year period were given a value of 1, and those which had never contained a nest were given a value of 0 – see below).

Local habitat variables were recorded in December 2009 when dormice were hibernating (note: these habitat variables were recorded by the lead author of this paper, RLW, not the volunteers, such that there was no scope for inter-observer variability). The number of trees and shrubs, and the plant species present, were recorded during a five-minute search within a 10 m radius of each nestbox to give an indication of the overall complexity and species diversity. Percentage ground cover was not calculated as cover varied greatly throughout the year. Data were collected during winter to better assess structural complexity related to tree branches. This provided a more meaningful value for this study than if foliage was dense, because dormice travel on branches, not leaves. Bird and small mammal nestbox data were obtained from the historic volunteer records (Table 1). Bird and small mammal nests were always removed from one year to another (bird nests were removed soon after young had fledged from the nest), and individuals were rarely found in a nest during the surveys. Consequently, nests were thought to be a more reliable indicator of bird or small mammal presence in a given year, so this variable was used in all analyses, instead of occupancy (as described for dormice in the previous section).

**Table 1:** Variables measured at each nestbox.

Measurement	Units and Further Information
Small mammal and bird nests Circumference of the nestbox tree	Percentage of occasions when nests were found in each box over 18 years As above (cm, measured at the height of the nestbox)
Distance of the nestbox from ground*	(m)
Angle of the nestbox floor*	Degrees from horizontal
Accessibility	Number of branches directly touching the nestbox
Distance from the edge of the woods	(m)
Distance from the nearest footpath	(m)
Distance from the stream (Kilcott Brook)	(m)
Number of trees in a 10 m radius	Trees were defined as plants taller than chest-height
Number of shrubs in a 10 m radius	Shrubs were defined as plants below chest-height
Woodland management regime*	Age of the coppice coupe in which the nestbox was situated (Obtained from the Gloucestershire Wildlife Trust)
Canopy cover	(%)
Canopy clumpiness	Index of dispersion value indicating the aggregation of the canopy
Mean structural complexity	(%) mean taken from two photos (see Methods for details)
Structural complexity clumpiness	Index of dispersion value indicating the aggregation of the shrub layer
Moss (Bryophyta)	Presence or absence in 10 m radius (1 = present; 0 = absent)
Ash ( *Fraxinus* *excelsior* )	As above
Bramble ( *Rubus* *fruticosus* agg.)	As above
Pedunculate oak ( *Quercus* *robur* )	As above
Honeysuckle ( *Lonicera* *periclymenum* )	As above
Ferns (Pteridophyta)	As above
Dog’s mercury ( *Mercurialis* *perennis* )	As above
Holly ( *Ilex* *aquifolium* )	As above
Hawthorn ( *Crataegus* *monogyna* )	As above
Hart’s-tongue ferns ( *Asplenium* *scolopendrium* )	As above
Ivy ( *Hedera* *helix* )	As above
Grasses (Poeace)	As above
Sycamore ( *Acer* *pseudoplatanus* )	As above
Crab apple ( *Malus* *sylvestris* )	As above
Other vegetation	As above

Hazel (a dominant species in the wood) was excluded as it was always found within 10 m of every nestbox. Other vegetation refers to plants growing from the ground. For details of canopy cover, clumpiness and structural complexity parameters, see Methods. * Angle of the nestbox floor and distance of the nestbox from the ground were not included in the analysis because these varied when nestboxes were handled during dormouse monitoring surveys and would not, therefore, be consistent over time. Woodland management regime was also disregarded because several coppicing dates could not be determined.

To record canopy complexity and structural complexity of the surrounding shrub layer, three photographs were taken at each nestbox, one vertically upward and two horizontally at nestbox height (these standard images were taken using a Canon IXUS 860 IS compact digital camera rather than hemispherical images taken with a fish-eye lens, so picture distortion did not need to be accounted for [34]). The shrub layer photographs, one behind and one in front of the nestbox, were taken against a white sheet for contrast. Vegetation density and complexity were calculated using CanopyDigi [34]. This digital image analysis provided an objective quantification of vegetation cover and an index of dispersion value to assess vegetation aggregation and identify significant gaps (high values = clumping with gaps; low values = more uniform vegetation – [35]. Shrub layer structural complexity was calculated using the mean of the two photographs, creating a mean percentage cover and mean index of dispersion.

### Statistical methods

To test whether actual nestbox occupation data showed significant departures from a random distribution, as expected if nestboxes were actively chosen but not if they were randomly selected, the frequency of dormouse occupation in each nestbox over the 18 years was compared to a hypothetical Poisson distribution. This was done using a Kolmogorov-Smirnov test (as per [36]).

All percentage variables were converted to proportions and arcsine transformed. Given that the circumference of trees would have increased over the study period, values in this variable were ranked (1 = smallest circumference) rather than using absolute values. The age of coppice, angle of nestbox floor and height of nestbox were not included in the analysis because coppice dates were not known for all sections of the wood, and the height and angle of the nestbox would have changed during the monthly surveys as the volunteers monitored the contents of the nestboxes.

A stepwise regression was used to determine which independent variables were predictors of dormouse nestbox selection, using both forward and backward procedures (the default for the ‘step’ command in the R statistical software package) and Akaike’s Information Criterion (AIC) as a method of model reduction. This allows the optimal (sub) set of predictors to be identified and maximum parsimony to be achieved. This analysis used the percentage of dormouse occupancy over the 18-year period as the dependent variable. Standardised residuals of the final regression were normally distributed, as verified by a Lilliefors test for normality (D = 0.08, p = 0.12) (as per [37]).

To remove any bias that could have arisen from dormice reselecting nestboxes in which nests remained from one year to another, a stepwise binary logistic regression was run (1 = nestbox containing a nest sometime during the 18-year period; 0 = never occupied by dormice). Note that the number of nestboxes each year remained the same (n = 97). The independent variables of small mammal nests and bird nests were still percentages (as above) because these nests were always cleared out from year to year, thus removing any confounding effects. The logistic regression was more robust to the assumptions of the data than the use of percentage occupancy over 18 years. This conversion to simple presence or absence of a nest in the entire 18 year period also lost valuable information on the preference of nestboxes, i.e. a box occupied once in 18 years was given the same value as a box occupied in most years. Given that our aim was to understand factors influencing nestbox selection, this detail of preference was useful. Similar results from both analyses would strengthen the evidence that significant factors were of biological importance.

To further investigate the relationship between bird nests and dormouse occupancy within years (this was found to be significant in the first stepwise model – see Results), a Spearman’s Rank correlation was run comparing the percentage of dormouse occupancy and bird nests for all nestboxes together over each individual year. Finally, possible competitive effects between dormice and birds were examined between individual nestboxes, in each individual year. The percentage of cases where dormouse and bird nests were found (along with percentage of cases where only bird nests, only dormouse nests, or neither of these, were found) were compared against expected values calculated by the equation:

p(D|B) = p(B|D) = B * D, 

where the probability of dormice being found when bird nests were present is equal to the probability of bird nests being found when dormice were present at any point during the year (i.e. when no facilitation or competition is occurring), and B is the average percentage of bird nests found in all nestboxes over all years, and D is the average percentage of dormice found in all nestboxes over all years. Differences between expected and observed values were tested with a chi-squared test.

### Ethics statement

This study was conducted on publicly accessible land owned by Gloucestershire Wildlife Trust, who were aware that the study was being undertaken. No specific permissions were required to access the land or to undertake the study. Dormice are a protected species in the UK, requiring a handling license if they are being disturbed, however, no dormice were handled during the collection of habitat data for this study: these data were collected during winter when dormice were hibernating, thus ensuring that dormice were not disturbed. Occupancy data were collected before the study began, as part of a national monitoring programme, by trained volunteers with dormouse handling licenses. Dormice were put back inside their respective nestboxes promptly after the necessary data were recorded. No dormice were harmed during this procedure. licenses were granted only after volunteers had proven that they could handle dormice safely without harming them. For the purpose of this study, the lead author (RLW) also obtained a dormouse handling license issued by Natural England to undertake the work (license number 20121036); the conditions of this license were observed at all times. See [33] for further information.

## Results

Occupation of nestboxes was relatively low, with an average of 7.3% of boxes occupied in any given year (S.D. = 3.3, minimum 2%, maximum 13%). Occupation of nestboxes was not random (Z = 5.07, n = 97, p < 0.01), indicating active nestbox selection. The final stepwise-reduced model was highly significant (F_8,88_ = 5.68, p < 0.01) and the suite of habitat variables entered explained 28% of variability in dormouse occupancy (adjusted r^2^ = 0.28) (Table 2). It is important to note that the stepwise approach creates a best-fit model of numerous predictor variables in a multivariate framework, balancing model explanatory power and parsimony. Overall, this model is highly significant, and all explanatory variables in the model are important in achieving the overall significance and R^2^ value, and warrant further discussion. Not all explanatory variables are independently significant in this final model (Table 2) since many of these are important in association with other variables (i.e. there is no simple univariate relationship). The most important factor determining occupancy was the distance from the wood edge. This was a positive correlation, indicating that dormice preferred nestboxes towards the centre of the wood. There was a negative relationship between occupancy and the circumference of the nestbox tree; smaller trees were associated more strongly with nestbox use than larger trees. There was also a negative relationship with the presence of ferns. Presence of oak and canopy clumpiness, as well as the number of trees and the presence of hawthorn were also important factors in the final best-fit model.

**Table 2:** Variables found to be important for dormouse nestbox selection, as determined by a stepwise regression.

	df	AIC	Delta AIC	Relationship	p-value
Hawthorn	1	-312.4	0	Negative	p = 0.14
Number of trees	1	-311.9	9.6	Positive	p = 0.11
Oak	1	-311.5	11.7	Positive	p = 0.08.
Canopy clumpiness	1	-311.1	13.2	Positive	p = 0.07.
Ferns	1	-310.4	13.9	Negative	p = 0.05*
Birds	1	-308.9	14.3	Positive	p = 0.02*
Circumference of the tree	1	-306.8	14.7	Negative	p < 0.01**
Distance from edge of wood	1	-297.2	15.2	Positive	p < 0.01***

Significance codes: ‘*** ’ p < 0.001 ‘** ’ p < 0.01 ‘* ’ p < 0.05 ‘.’ p < 0.1

**Figure 1:**
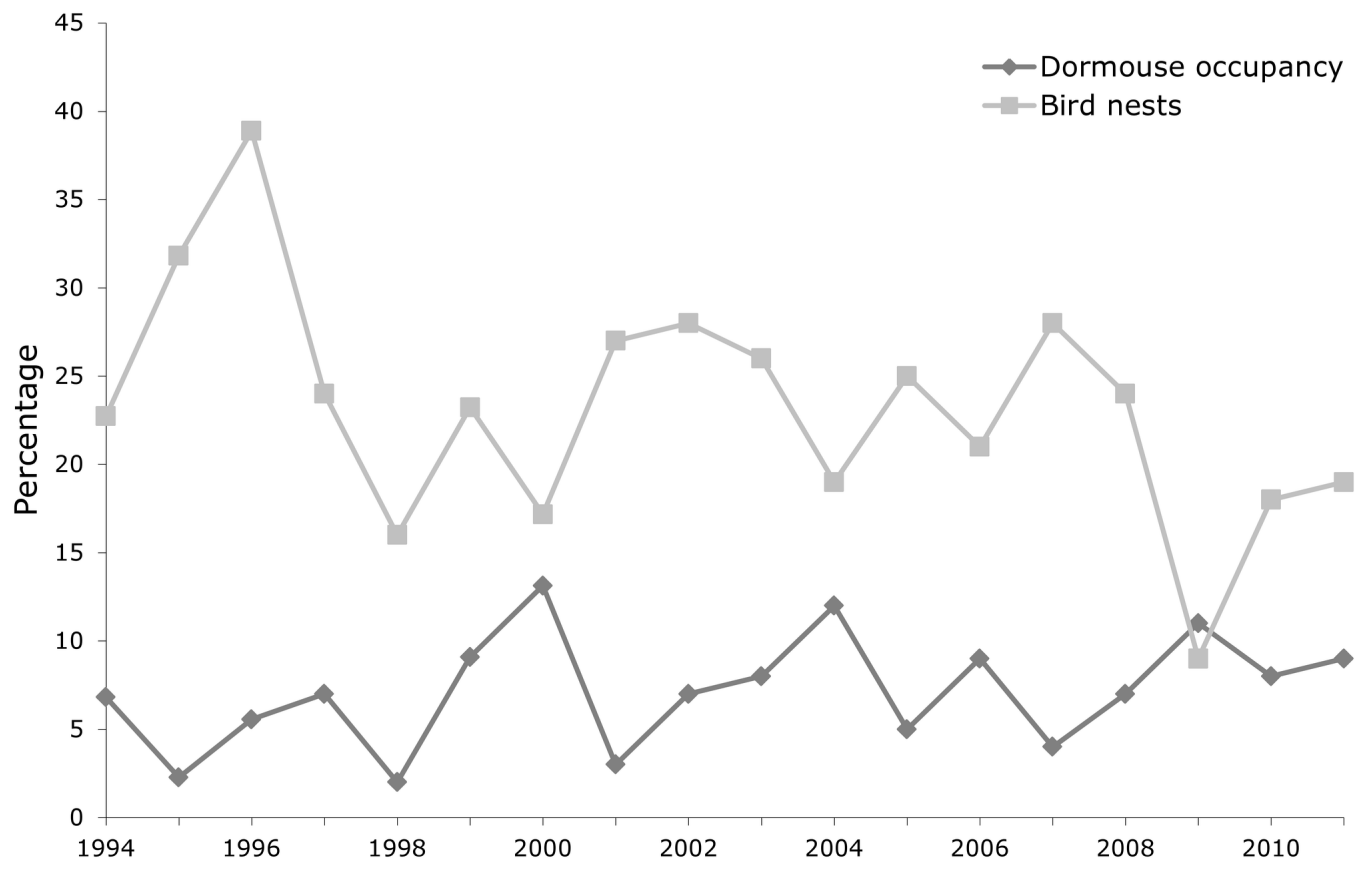
Percentage of nestboxes containing bird nests and dormice (individuals rather than nests) between 1994 and 2011.

The stepwise regression also showed that there was a positive relationship between dormouse occupancy and bird nests (Table 2), indicating: (1) no evidence of competition in the study population and (2) that nestboxes were selected on the basis of similar, or at least closely correlated, variables. When bird nests and dormouse occupancy were further examined for all nestboxes within years, a relatively strong significant negative correlation was found (r_s_ = -0.56; n = 18; p = 0.016), implying potential competition or mutual exclusion on a yearly basis (Figure 1). Comparison of the observed percentage of occupation of each nestbox in a given year by birds, dormice or both showed no significant difference to calculated expected values where dormouse and bird occupation were calculated independently of one another (χ^2^ = 0.01; df = 3; p > 0.99), hence, there was no evidence of competition between birds and dormice at this site.

In the stepwise binary logistic regression, five factors were found to be significant and these explained 28% of dormouse nestbox selection in total (estimated R^2^ = 0.28, Wald = 0.83, p < 0.01). Distance from the edge of the wood remained the most significant explanatory variable (p < 0.01), followed by canopy clumpiness (p = 0.03). Ferns, honeysuckle and sycamore were also important in the final best-fit model (Table 3).

**Table 3:** Variables found to be important for dormouse nestbox selection, as determined by a binary stepwise logistic regression.

	df	AIC	Delta AIC	Gradient	p-value
Honeysuckle	1	122.9	0	Positive	p = 0.16
Ferns	1	124.4	1.5	Negative	p = 0.07.
Sycamore	1	125.8	2.9	Negative	p = 0.10
Canopy clumpiness	1	126.2	3.3	Positive	p = 0.03*
Distance from edge of wood	1	129.5	6.6	Positive	p < 0.01**

Significance codes: ‘** ’ p < 0.01 ‘* ’ p < 0.05 ‘.’ p < 0.1

## Discussion

This study demonstrates three main points. Firstly, dormice actively select nestboxes, a point often overlooked or impossible to test in habitat selection studies (e.g. [36,38]). Secondly, a suite of habitat factors can explain a considerable degree of this nestbox selection, which could inform the placement of nestboxes for the purpose of dormouse research and conservation. Thirdly, volunteers can collect useful data on dormouse nestbox occupation.

Several of the factors included as candidate variables in the model influenced dormouse nestbox selection, together explaining 28% of variability in occupancy. The most influential factor was the distance from the edge of the wood, which may be due to edge effects (such as increased predation or competition [39]), although some nestboxes were occupied despite being close to the wood edge and the presence of potential dormouse predators was not recorded in this study (for example, corvid birds are potential predators of dormice in edge habitats – see 40. As Midger Wood is a small wood (9 ha), it is not possible to determine at what point distance to the edge of the wood would cease to be important, for example, in a much larger wood. Additionally, the shape of the woodland might affect the importance of the distance to edge variable on dormouse nestbox selection, since this affects the edge:interior ratio. The influence of edge effects on nest-site selection has been studied mainly in avian populations [41] and there are currently no studies on its effect on dormice, although edge effect influences in smaller woods have been proposed [42]. Contradictory results show that dormice readily occupied nest tubes on the fringe of dense scrub in Dorset (S. Eden, pers. comm.), possibly because these were less favoured by competing small mammals (note: nest tubes consist of a length of corrugated plastic tubing and square in section containing a sliding wooden tray [43]). There may be very different selection pressures influencing populations across different habitats.

Since dormice selected nestboxes on thinner trees in this study, it may be that larger trees supported more natural nest-sites such as cavities or dense foliage in the canopy. There is much contention as to whether dormice prefer to use nestboxes or natural nest-sites and factors vary greatly in different habitats. In young woodlands, hedgerows and scrub, dormice may favour unenclosed natural nest-sites (e.g. woven into bramble) over tree hollows or artificial nest-sites [16,44], although in diverse, low-growing woodlands, radio-tracked dormice preferred nestboxes over natural nest-sites [20]. In coppice-with-standards woodland, radio-tracked dormice spent the majority of time either in nestboxes (34% of dormouse tracking days) or in natural tree hollows (41%) and far less time in natural nests in bramble (8%) [19]. Juškaitis [22] found that dormouse nestbox occupation was negatively, but weakly, correlated with tree crown density; the positive relationship with canopy clumpiness found in this study might be due to similar reasons, as gaps in the canopy would mean fewer arboreal routes, which may cause dormice to descend to nestboxes. High canopy clumpiness meant that there were areas of dense cover but also large gaps that let through direct sunlight, which would benefit the plant species that dormice use for food and nest material. It is still unclear how selection for natural nest-sites interacts with nestbox selection mechanisms, and this would be an interesting area for further investigation. Note that studies into natural nest-sites in woodland are facilitated by radio-tracking, and this is unlikely to be feasible using NDMP volunteers due to the legislation surrounding fitting radio-tracking devices to dormice, and the prohibitive costs involved. Nestboxes therefore remain a more practical way of studying dormice with the help of volunteers.

The presence of certain plant species influenced dormouse nestbox selection: dormice were positively correlated with oak and honeysuckle, and negatively correlated with ferns, sycamore and hawthorn. Food sources influence nestbox selection, as dormice rarely travel further than 100 m from their nests but require a diversity of food sources to ensure that food is available continuously throughout the active season [12,20]. Honeysuckle and oak are important food sources [12,18,45,46], with honeysuckle also forming an important component of dormouse nests in Midger Wood [30]. It is therefore unsurprising that these plant species are important explanatory variables in the final models. The presence of ferns is characteristic of dark and damp areas [47], which may be avoided by dormice. The negative relationship with sycamore is unlikely to be biologically meaningful as this species was only present near three nestboxes (these never contained dormice, and this is the reason for its statistical inclusion in the stepwise regression).

The lack of competition between dormice and other nestbox inhabitants was of particular interest in this study because competition for nestboxes occurs in other studies (e.g. [22,48]). Although the lack of competition between birds and dormice agreed with the findings of Morris et al. [24]), years in which dormice occupied more nestboxes generally coincided with years in which birds occupied fewer nestboxes, implying that larger scale effects such as population fluctuations might influence nestbox occupancy. The amount of volunteer-collected data available on birds and dormice might provide an opportunity to investigate this relationship further.

The remaining variability in our study might be explained by chance, variables that were not measured as part of this study (e.g. climate, predators, parasites, pathogens etc), or by dormouse learning and previous experience. Indeed, Marsh and Morris [49] found that boxes favoured by dormice in one year tended to be reselected by them in the following year; however, since individuals were not individually marked for identification at the study site, it was not possible to investigate this. Furthermore, since our study only investigated one small woodland in the UK, it is possible that the results may be site and size specific, and further exploration would be needed to elucidate the generality of the results. The temporal span of the dataset was 18 years, and some of the explanatory factors may have changed over this time despite consistent management by GWT. The influence of parasites and predators on dormouse nestbox selection would be an interesting topic for future study, but as this would require annual records of the relevant variables, it was not possible to examine this here using a historical dataset.

Using volunteer-collected data has both advantages and disadvantages. Alongside the usual benefits of saving time and money compared to recruiting professionals [e.g. 27, 28], a key advantage of this volunteer-collected dataset was its longevity; this can also be an important attribute of useful volunteer-collected data [50]. Additionally, volunteers surveyed the nestboxes monthly from April to November, the highest recommended number of nestbox checks in a year [51]. As a result of this, the dataset was large (>30,000 data points: 97 boxes * 6 months * 18 years * 3 species – dormice, small mammals and birds), which reduced the chance of a type II error.

Volunteer-collected data also has indirect benefits. For example, volunteers also monitored any issues at the site (e.g. fallen trees across footpaths) and reported these back to GWT, thus facilitating the overall management of the site. Most of the regular volunteers at Midger Wood were members of GWT, providing financial support through their memberships and therefore contributing to the cost of managing the site as well as collecting data. Newman et al. [27] found that at least 30% of their volunteers joined conservation organisations after they had volunteered on their project. Meaningful interactions with the natural world also have the potential to enhance human wellbeing and quality of life [52,53] and volunteers who participated in mammal surveying projects gained fulfilment and knowledge [27]. When asked, volunteers at Midger Wood stated that they gained enjoyment from monitoring the nestboxes and some had been participating in dormouse surveys at the site for 18 years.

The volunteer dataset did, however, present some analytical challenges. Although nestbox occupancy data were collected regularly and followed the majority of NDMP guidelines, volunteers did not collect any habitat data, despite habitat data being requested at 5-year intervals for the NDMP. These habitat data would have proved extremely useful in the present study. Habitat characteristics were measured by the authors at the end of an 18-year period, and we were aware that some of these would have changed during this time. A careful analysis and a consideration of variables that may have changed resulted in useful trends being identified. Some nestbox records were difficult to interpret and, if they could not be confirmed, they had to be discarded from the dataset (<5% of the records). There was a certain degree of variability in the records that made computerising the dataset time-consuming (e.g. a record of “*DORMOUSE*” was described as an unoccupied dormouse nest at the bottom of the recording form, not the presence of a dormouse as suggested). Exact records (i.e. how nestbox contents were recorded on the form) were variable between different people despite using the same data recording forms, and this issue increased with the fluctuating number of volunteers.

## Conclusions and Recommendations

This study has developed work by previous researchers and has furthered understanding of dormouse nestbox selection. It indicates that dormice select nestboxes based on a combination of factors. While views on the importance of nestboxes for dormouse conservation differ, many, but not all of the results of this study are likely to be relevant for natural nest selection too. Large scale features, such as distance to the edge of the wood, or combinations of plant species in the nearby vicinity are likely to apply equally to natural nests and nestboxes. Some localised factors may differ, as nestboxes provide shelter that may be absent on thin trees with low structural complexity, which would prevent dormice from building natural nests on these trees. Nevertheless, these results are important in informing conservation management decisions where nestboxes are used, and, in combination with other studies, in understanding the broad principles of dormouse habitat selection in any woodland.

Monitoring dormice using volunteers can provide an adequate quantity of analysable data, and useful information can be extracted from data that might usually be considered less reliable compared to rigourous scientific data, as shown in other studies (e.g. [50,54]). Volunteer schemes with large historical datasets are irreplaceable and invaluable as they can produce important ecological information and can help identify important sites and management strategies [28,54]. NDMP records vary greatly in quantity and quality between sites and years (S. Sharafi, PTES, pers. comm.), so it would be useful to determine the reasons behind this variation in order to uncover ways in which to reduce it, thus improving the national database. Volunteers should be informed of the importance of completing forms consistently and of collecting regular habitat data, and guidance on this matter should be given to the leaders of monitoring groups. Volunteer schemes would undoubtedly benefit from scientific input to improve data collection, thereby facilitating scientific study of those data and allowing the results to be of maximum usefulness for applied ecology and conservation.
